# Dispersion of atmospheric air pollution in summer and winter season

**DOI:** 10.1007/s10661-017-6319-2

**Published:** 2017-11-04

**Authors:** Robert Cichowicz, Grzegorz Wielgosiński, Wojciech Fetter

**Affiliations:** 1 Faculty of Architecture, Civil and Environmental Engineering, Lodz University of Technology, Al. Politechniki 6, 90-924 Lodz, Poland; 2 Faculty of Process and Environmental Engineering, Lodz University of Technology, Wolczanska 175, 90-924 Lodz, Poland; 3 Department of Environmental Protection of Pątnów-Adamów-Konin Power Complex S.A, Kazimierska 45, 62-510 Konin, Poland

**Keywords:** Dispersion, Summer and winter season, Air quality

## Abstract

Seasonal variation of air pollution is associated with variety of seasons and specificity of particular months which form the so-called summer and winter season also known as the “heating” season. The occurrence of higher values of air pollution in different months of a year is associated with the type of climate, and accordingly with different atmospheric conditions in particular months, changing state of weather on a given day, and anthropogenic activity. The appearance of these conditions results in different levels of air pollution characteristic for a given period. The study uses data collected during a seven-year period (2009–2015) in the automatic measuring station of immissions located in Eastern Wielkopolska. The analysis concerns the average and maximum values of air pollution (i.e., particulate matter PM10, sulfur dioxide, nitrogen dioxide, carbon monoxide, and ozone) from the perspective of their occurrence in particular seasons and months or in relation to meteorological actors such as temperature, humidity, and wind speed.

## Introduction

Air quality in both Poland and other countries of the world depends on the amount of pollutant emissions, the intensity and type of physicochemical changes taking place in the atmosphere, and the large-scale movements of polluted air masses. Atmospheric air is an element of the natural environment for which no natural protective barriers can be isolated, and therefore, the control and analysis of the impact of particular pollutants not only on a global but also continental, national, and local scale are essential (Cichowicz and Wielgosiński 2015a, b; Ménard et al. 2016; Vallero 2014). The problem of air quality was noted by the World Health Organization (WHO), which estimated in 2012 that about 7 million deaths were associated with living in the areas with polluted air. The most important legislative act defining the requirements for air protection for all EU member states is Directive 2008/50/EC of the European Parliament and Council of 21 May 2008 on air quality and cleaner air for Europe called Clean Air for Europe (CAFE). Its aim is to improve air quality and protect against the harmful effects of pollution on the environment.

Sources of air pollutants are mainly combustion processes, various technological processes as well as vehicle traffic (Cichowicz and Wielgosiński 2015a, b; Gurney et al. 2012; Lelieveld et al. 2015; Nemitz et al. 2002). It should be borne in mind at the same time that low-emission sources emit pollutants primarily during the heating season, and that remote systems do it with varying intensity throughout the entire calendar year (Cichowicz and Wielgosiński 2015a, b; Lin et al. 2011).

Since the emitted pollutants are subjected to both dispersion and advection in the air, the analysis of such phenomena should take into account both the wind speed and direction, vertical movements of air due to thermodynamic equilibrium of the atmosphere, and local turbulences caused by altitude contrasts in the land cover. As a result of the dispersion of pollutants emitted to the air, the determined concentrations of pollutants are formed on the Earth’s surface, which are related to the limit values specified in national and international law (Colls 2002).

## Experimental

**Fig. 1:**
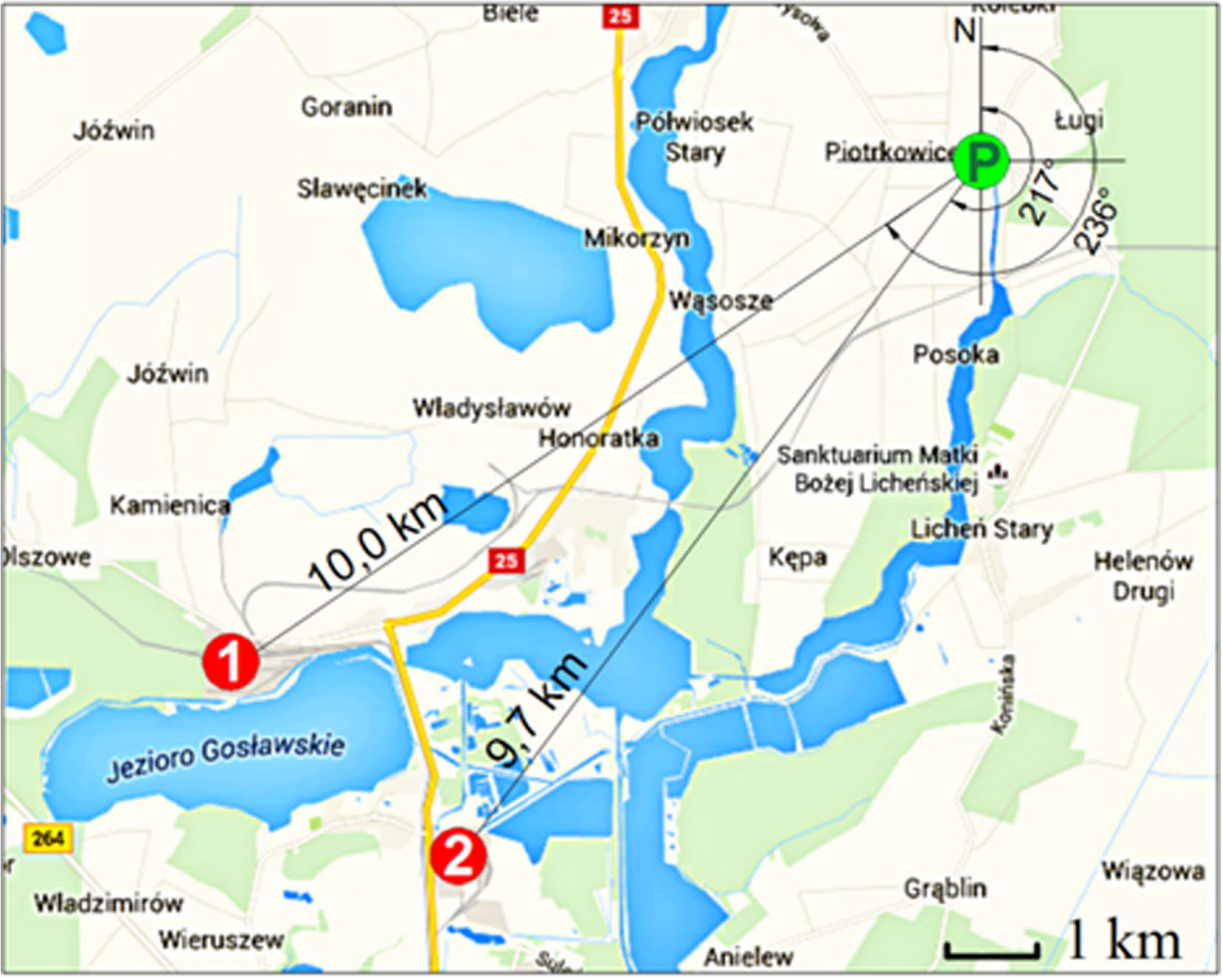
Location of the monitoring station Piotrkowice (P) in relation to the Pątnów Power Plants I, II (1), and Konin Power Plant (2) (https://www.google.pl/maps/@52.3539092,18.3324928,13.64z?hl = pl)

The analyses used data from a seven-year period covering the years 2009–2015, originating from an automatic atmospheric air monitoring station, located in the eastern part of Wielkopolska, in the Konin district, in the municipality of Ślesin in Piotrkowice (Fig. 1). The Konin district covers an area of 1578.7 km^2^ with a population density of about 82 persons/km^2^. The surrounding area is primarily arable land and meadows. The nearest human settlements are located in the following distances: 0.6 km—Piotrkowice village (532 inhabitants), 1.5 km—Wygoda village (270 inhabitants), 2.1 km—Niedźwiady Duże village (163 inhabitants), and 3.1 km—Półwiosek Stary village (263 inhabitants) as well as about 3.5 km—town of Ślesin (about 3200 inhabitants) and 4.2 km—town of Licheń (about 1500 inhabitants). The natural resource of the region is primarily lignite, whose mining has been taking place in the northern part of the region for over 70 years and is used as fuel in power plants “Pątnów I”, “Pątnów II”, and “Konin”, which are parts of the Pątnów-Adamów-Konin Power Plant Complex—ZE PAK S.A.

To illustrate the hypothetical scale of environmental impact of the plants, the average amount of combusted lignite, biomass, fuel oil, and the amount of electricity and heat produced in the analyzed period is presented in Tables 1 and 2. This was compared with the mean annual concentrations of pollutants emitted.

**Table 1:** Production of electricity and heat—annual values from 2009 to 2015 (ZE PAK S.A. corporate materials)

Year	Electric energy production	Heat energy production	Coal consumption	Mazut consumption	Biomass consumption
	GWh/year	TJ/year	Gg/year	Gg/year	Gg/year
Pątnów I Power Plant					
2009	5239	no data	6316	8222	108,662
2010	4480	106	5461	9360	149,204
2011	4859	151	6113	9929	77,203
2012	5140	153	6450	13,193	164,863
2013	5076	129	6576	13,772	36,710
2014	4501	146	5827	6934	0
2015	4532	178	6004	7074	0
Pątnów II Power Plant					
2009	2213	–	2180	1278	0
2010	2512	–	2478	1624	0
2011	2429	–	2447	849	0
2012	2807	–	2942	703	0
2013	2650	–	2782	1 295	0
2014	2558	–	2725	1184	0
2015	2228	–	2425	779	0
Konin Heating and Power Plant					
2009	675	1595	900	1385	217,863
2010	574	1750	841	973	219,395
2011	451	1458	674	383	118 223
2012	392	1496	673	621	24,448
2013	411	1497	765	376	0
2014	416	1340	801	298	0
2015	388	1314	713	459	0

**Table 2:** Emission of pollutants—annual values from 2009 to 2015 (ZE PAK S.A. corporate materials)

Year	TSP	SO_2_	NO_2_	CO
	Mg/year	Mg/year	Mg/year	Mg/year
Pątnów I Power Plant				
2009	459	8037	8230	780
2010	390	6262	6298	686
2011	456	7721	6674	384
2012	467	7499	7447	448
2013	650	6630	7429	330
2014	587	5240	7031	b.d.
2015	338	5199	6721	b.d.
Pątnów II Power Plant				
2009	92	1413	1413	220
2010	108	1012	1770	203
2011	108	1190	1755	242
2012	121	1518	2080	264
2013	116	1698	2101	259
2014	98	1337	1894	b.d.
2015	74	1149	1654	b.d.
Konin Heating and Power Plant				
2009	69	1222	1613	557
2010	71	1693	1388	520
2011	91	1061	1050	388
2012	81	1374	1129	277
2013	71	1304	1296	56
2014	86	1565	1312	b.d.
2015	69	1376	1382	b.d.

The air monitoring station is located in a 2.5 × 3.0 m container 2.5 m high, which is resistant to external weather conditions. The following pollutants have been measured: particulate matter PM10 (in concentrations ranging from 5.0 μg/m^3^ to 250 mg/m^3^), sulfur dioxide (0 to 500 ppb), carbon monoxide (0 to 50 ppm), nitrogen oxide (0 to 500 ppb), nitrogen dioxide (0 to 500 ppb), nitrogen oxides NO_x_, as the sum of NO and NO_2_ (0 to 500 ppb), and ozone (0 to 500 ppb). Thermo Scientific’s primary monitoring system was provided by the monitoring station. PM10 particulate matter measurement was carried out using a TEOM 1400a type analyzer based on a gas sample filtration system and automatic mass measurement. Sulfur dioxide concentrations were measured using an automatic Thermo 43i UV spectrometer. Nitrogen oxides (NO and NO_2_) are measured by a Thermo 42i chemiluminescent analyzer, and the carbon monoxide concentration is measured using an Thermo 48i IR spectrometer. Measurement of ozone concentration was provided by optical method at 254 nm (UV) using Thermo 49i analyzer.

All measurements of pollutants are made by analyzers whose measurement methods are in accordance with the reference methods specified in Annex VI of the CAFE Directive. Devices operate 24 h a day, only with small interruptions (about 1% of the time) dedicated to servicing and calibration.

Measurements of some meteorological parameters are also carried out at the station to determine the direction of flowing air masses and potential sources of pollution. They are made by means of two sensors that measure such air parameters as temperature (°C), relative humidity (%), wind direction (0–359°), and wind speed (m/s). The HMP 45A sensor produced by Vaisala is used to measure the temperature and humidity of the air. It was mounted in the meteorological radiation shield, while wind direction and wind speed measurements were carried out using a WindSonic ultrasonic sensor from GILL Instruments installed at the top of a 12-m meteorological mast.

## Results and discussion

For analysis, the results of measurements of air pollution and meteorological data recorded by the Piotrkowice measuring station in the seven-year period (2009–2015) were selected. It was assumed that the results of averaged individual measurements from the seven-year period would be analyzed. It was also assumed that the best way to express changes in concentrations recorded at the station would be to analyze their maximum values in terms of seasonality of emissions, including variations in recorded concentrations depending on direction and speed of wind and air temperature and humidity. Depending on the pollutant analyzed and its permissible values, 24-h, 1-h, or 8-h rolling averaging was used.

**Fig. 2:**
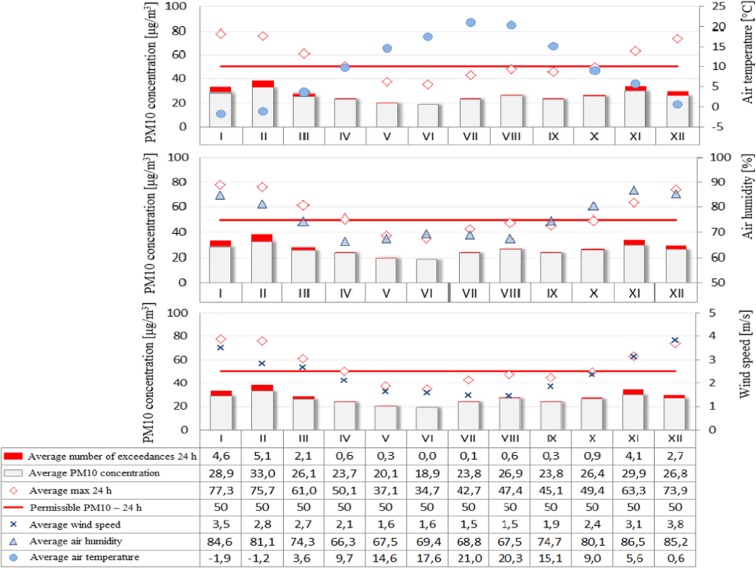
PM10 concentration depending on air temperature, humidity, and wind speed in particular months of the year

**Fig. 3:**
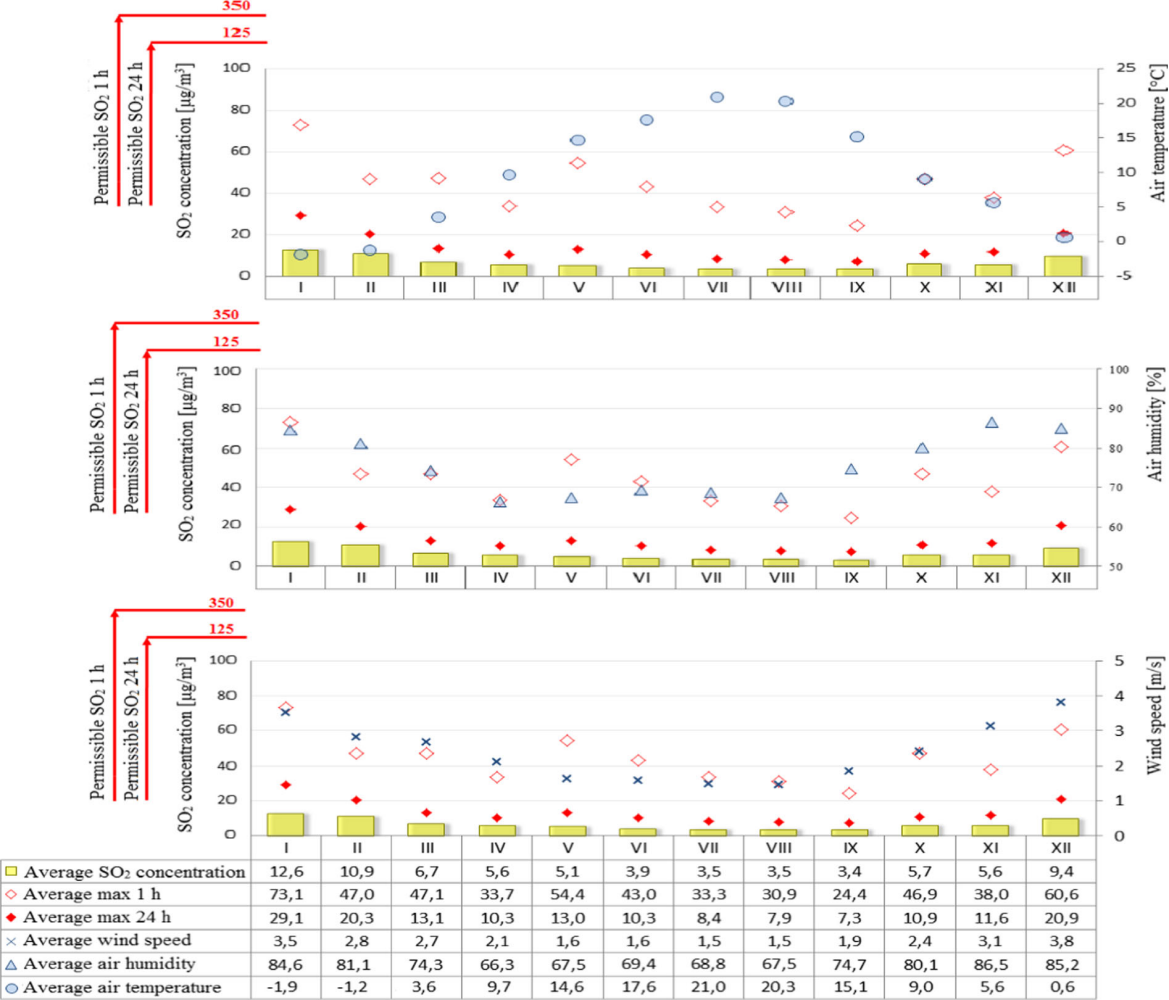
Sulfur dioxide concentration depending on air temperature, humidity, and wind speed in particular months of the year

**Fig. 4:**
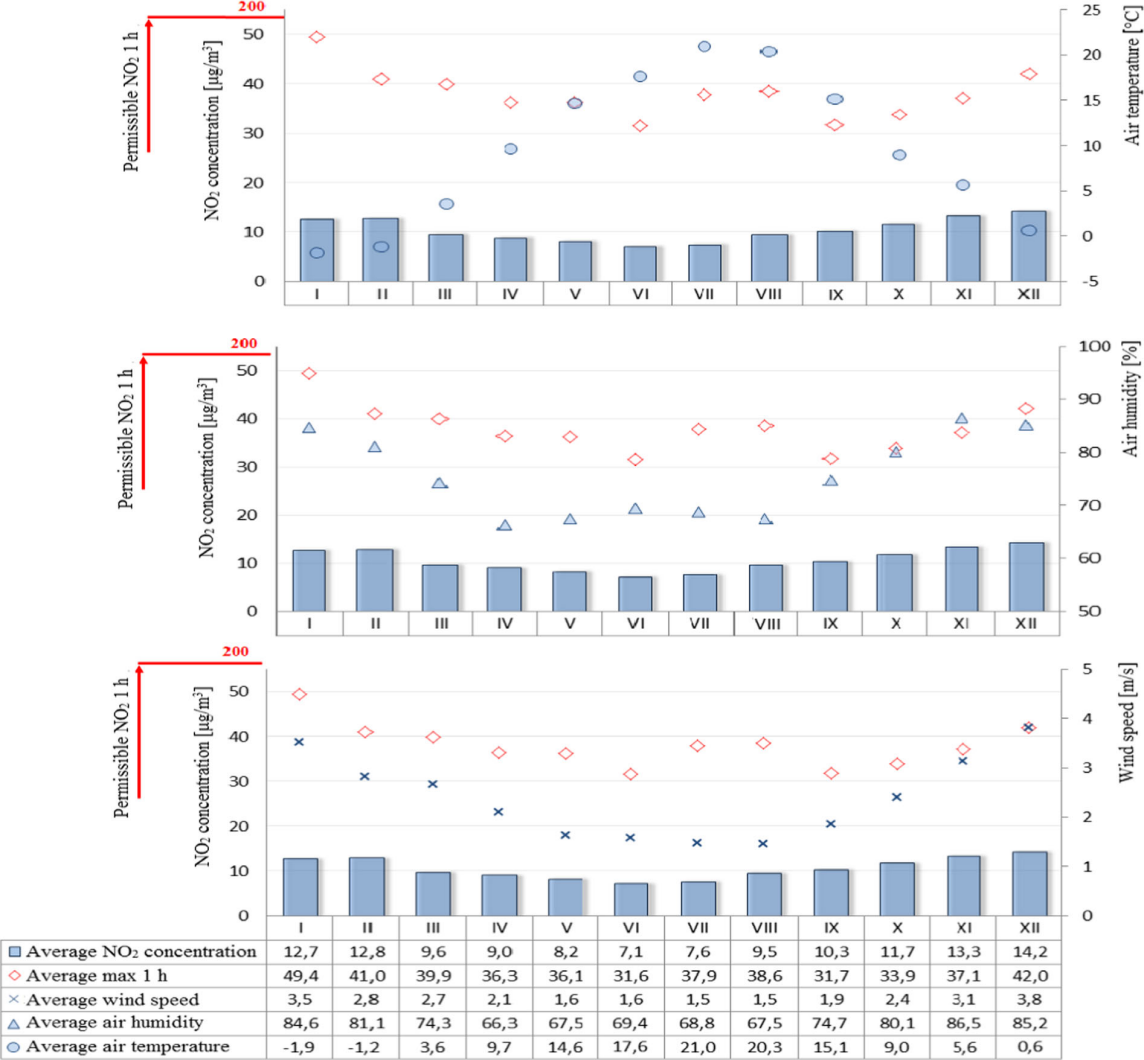
Nitrogen dioxide concentration depending on air temperature, humidity, and wind speed in particular months of the year

**Fig. 5:**
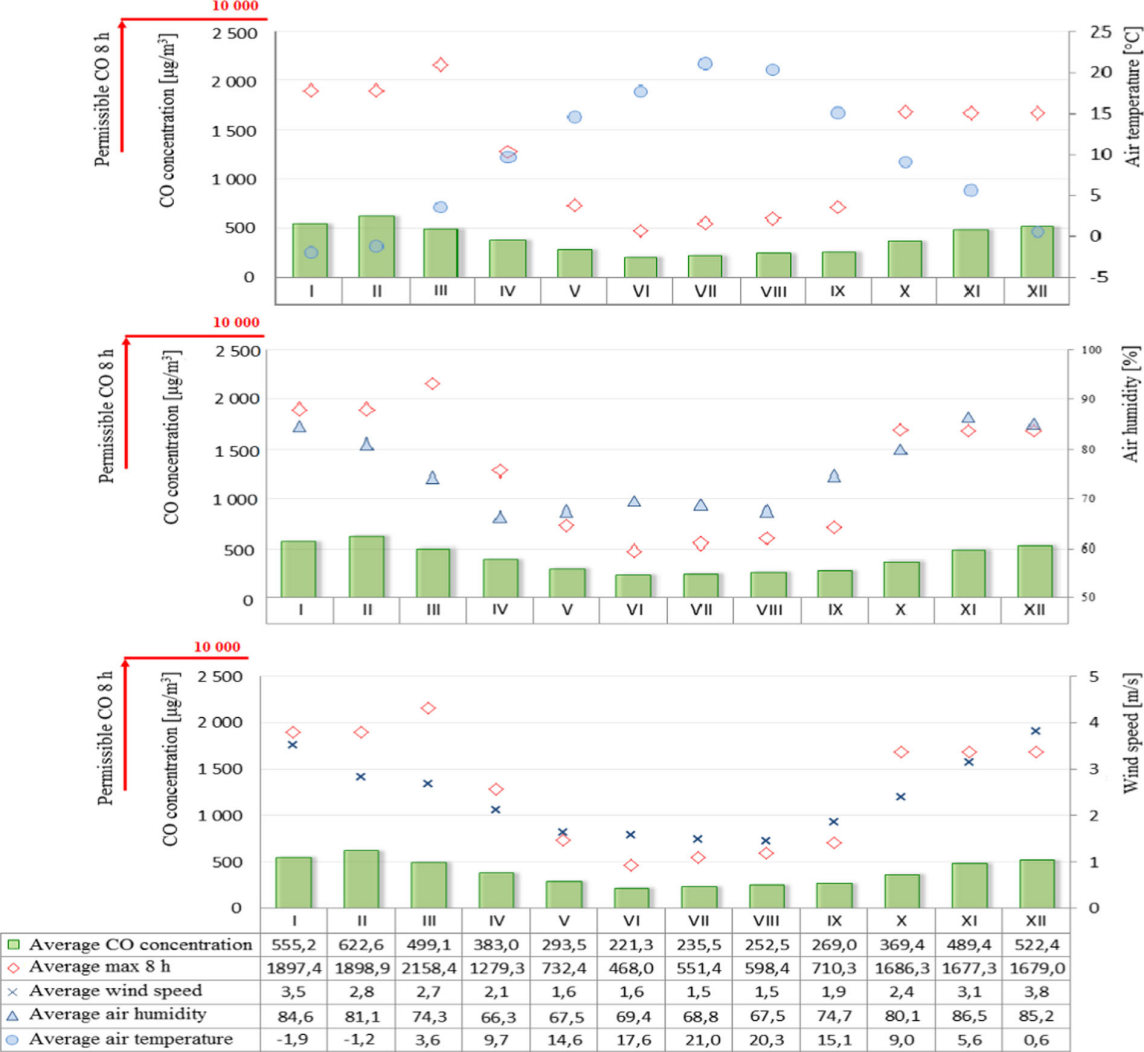
Carbon monoxide concentration depending on air temperature, humidity, and wind speed in particular months of the year

Figure 2 shows averaged daily concentrations of PM10 for both individual months and the average for the years 2009–2015. Similarly, Fig. 3 shows the average sulfur dioxide concentration for each month and the average for 2009–2015. On the other hand, the permissible level for nitrogen dioxide is the instantaneous (1 h) value of 200 μg/m^3^, so Fig. 4 shows the averages of the maximum instantaneous values from individual months in the seven-year period analyzed. A further analysis of the seasonality of air pollutants concerned carbon monoxide (Fig. 5), in which the recorded concentrations were referred to a permissible level of 10,000 μg/m^3^ and defined as an 8-h rolling average.

**Fig. 6:**
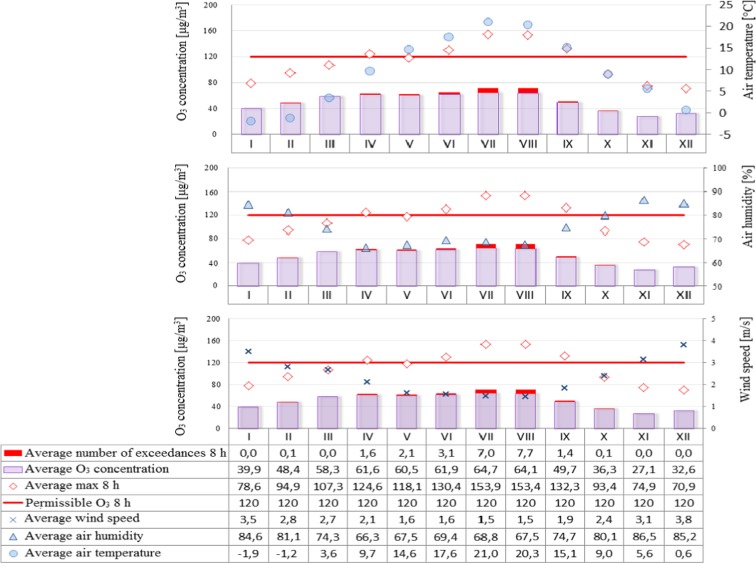
Ozone concentration depending on air temperature, humidity, and wind speed in particular months of the year

**Fig. 7:**
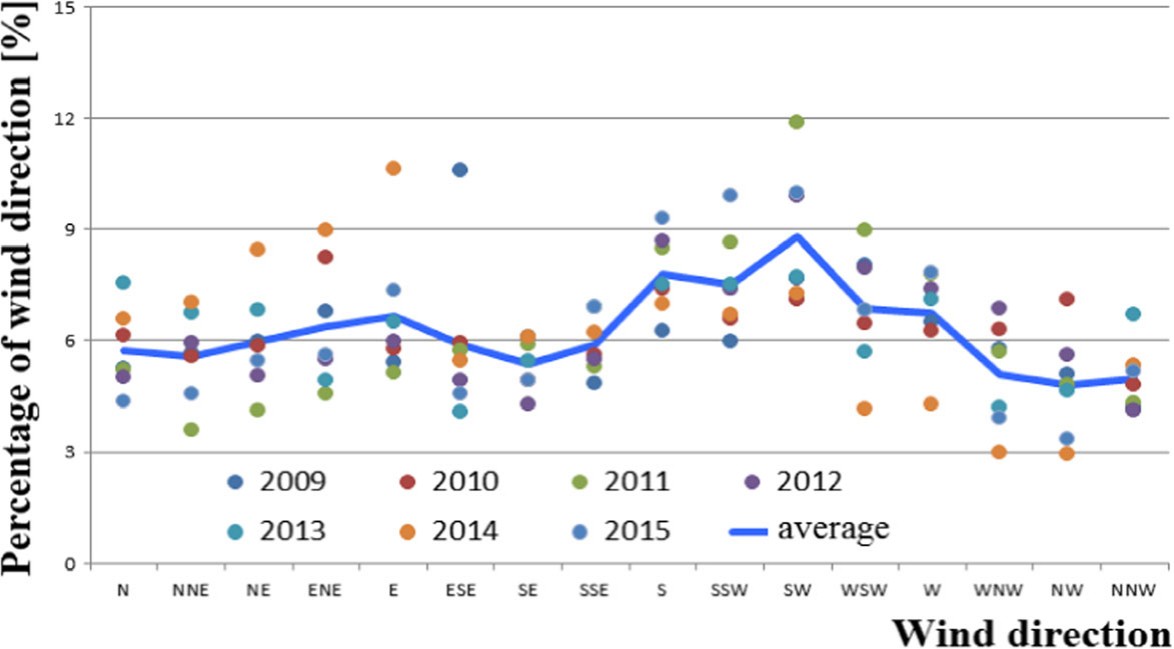
Percentage of particular wind directions from the years 2009–2015

**Fig. 8:**
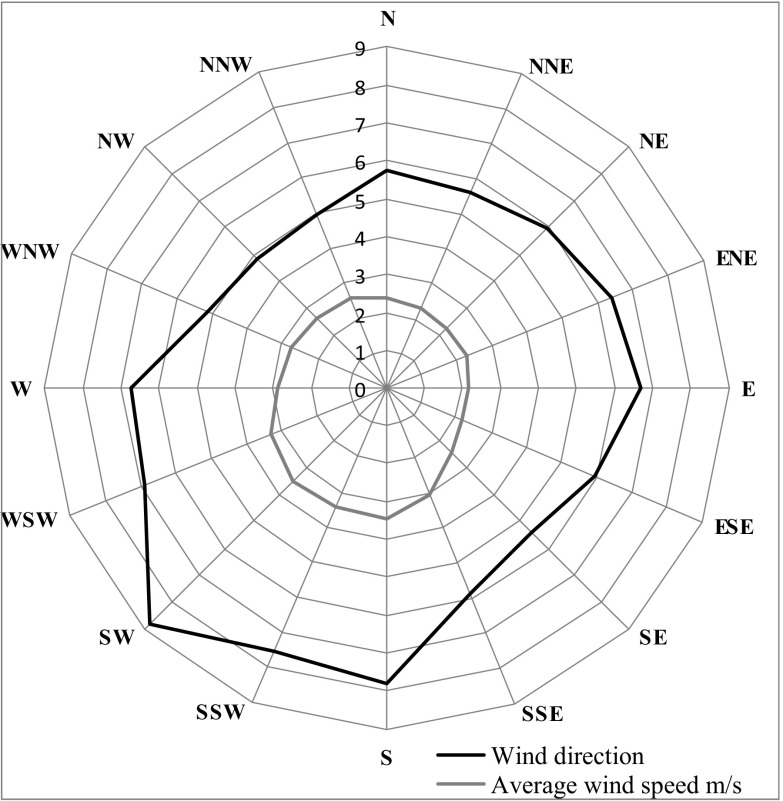
Wind rose for analyzed area (mean values for years 2009–2015)

**Fig. 9:**
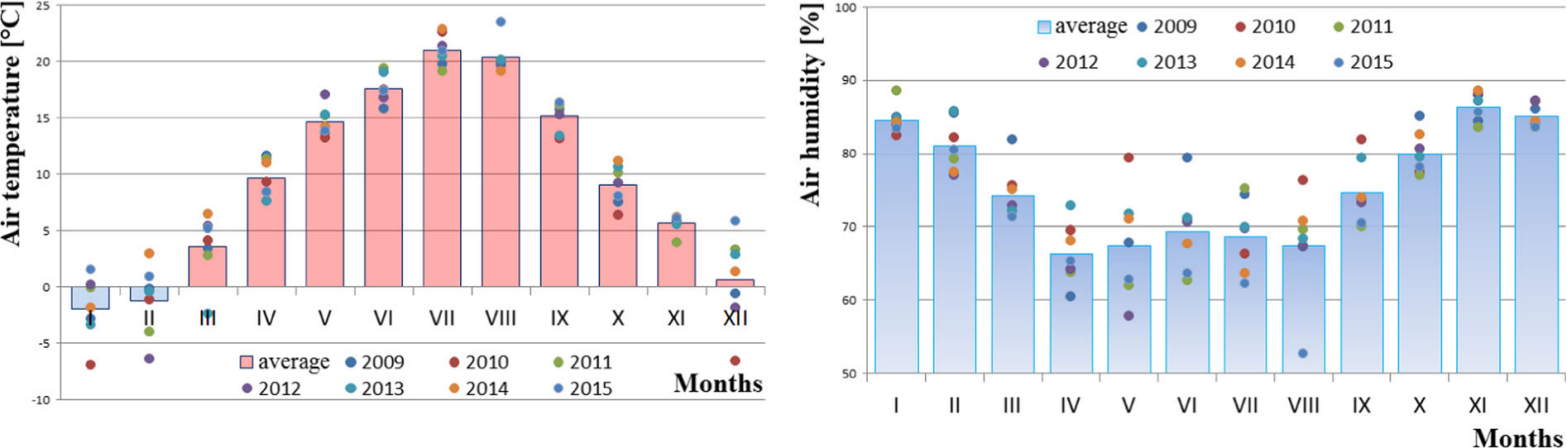
Average temperature [°C] and humidity [%] in months and years 2009–2015

On the other hand, the permissible level for ozone (Fig. 6) is 120 μg/m^3^, which is the maximum concentration that can occur on a given day, out of all 8-h rolling values of a given day. Figures 7, 8 and 9 show the analysis of meteorological conditions carried out to determine the direction of incoming air pollutants or to predict them. Of course, in order to be consistent with the results obtained for pollutants, meteorological data were analyzed for the same time period as for air pollutants.

## Conclusions

The analysis of air pollution in terms of seasonality can explicitly confirm the presence of elevated levels in winter months and during the heating season and low in summer months and during the summer season. An exception in this case is ozone whose specific formation in the lowest part of the Earth’s atmosphere causes high concentrations that appear in the periods of maximum insolation, longer days, and higher air temperatures, while low—when activity of the sun is lower, days are shorter, and air temperature is lower (Jeż 2009). Higher levels of air pollution in winter months may also be associated with increased low emissions from local home furnaces, as well as more frequent in these periods, inversion of temperature resulting in smog events. The higher levels of pollution in winter months are represented by PM10 concentrations which exceed the limit values. It was also observed that in winter the levels of such pollutants as sulfur dioxide, nitrogen dioxide, and carbon monoxide were increased but did not exceed the limit values. On the other hand, the reduced levels of such pollutants as nitrogen dioxide and carbon monoxide in summer months can be attributed to the contribution of these compounds to photochemical reactions occurring under the influence of solar radiation which result in the formation of ozone (Jeż 2009).

Considering the seasonal nature of air pollution, we should also refer to the recorded atmospheric conditions from the subsequent months of the analyzed period 2009–2015. It has been observed and confirmed that the values of temperature, humidity, and wind speed for individual months are comparable to those found in the “Atlas of Climate in the Wielkopolska Region” (Farat 2004).

A significant correlation was also found between the elevated levels of air pollutants (PM10, sulfur dioxide, nitrogen dioxide, and carbon monoxide) and low ozone levels in winter months and meteorological parameters such as air temperature (low values), air humidity (high values), and wind speed (high values) in the same time. On the other hand, in summer time, the opposite situation occurred, namely, when temperature was high and wind speed and humidity lower, air pollution was also reduced.
